# Report on personality and adherence to antibiotic therapy: a population-based study

**DOI:** 10.1186/2050-7283-1-24

**Published:** 2013-11-22

**Authors:** Malin Axelsson

**Affiliations:** Krefting Research Centre, Institute of Medicine, Internal Medicine and Clinical Nutrition Sahlgrenska Academy, University of Gothenburg, Gothenburg, Sweden; Department of Nursing, Health and Culture, University West, Trollhättan, Sweden

## Abstract

**Background:**

Antimicrobial resistance results from inappropriate use of antibiotics and makes common or life-threatening infections more difficult or sometimes impossible to treat. Proper adherence to antibiotic therapy is one among several measures required to prevent antimicrobial resistance. Knowledge of personality traits could help in identifying patients who need support with their adherence behaviour. Previous research has presented associations between personality traits and adherence to long-term medication treatment in individuals with different chronic diseases. However, there is limited knowledge about associations between personality traits and adherence to both antibiotic therapy and to shorter treatment periods. The aim was to explore the relation between personality and adherence behaviour in people prescribed antibiotics for common infections.

**Methods:**

In a population-based study, 445 respondents reported on their prescribed antibiotic therapy and completed the Neuroticism, Extraversion, and Openness to experience Five-factor Inventory and the Medication Adherence Report Scale. Data were statistically analysed using descriptive statistics, t-tests, bivariate correlations, multiple and logistic regressions.

**Results:**

Non-adherence was estimated to be 9.4%. The most common reasons for stopping therapy prematurely was that the respondent was now healthy and that the respondents experienced side-effects. Non-adherent respondents scored lower on the personality traits Agreeableness and Conscientiousness. A logistic regression showed that higher scores on Agreeableness decreased the risk for non-adherence to antibiotic therapy. In a multiple regression, Neuroticism was identified as a negative predictor, and both Agreeableness and Conscientiousness were identified as positive predictors of adherence behaviour.

**Conclusions:**

Preventive measures to decrease non-adherence may be to inform patients not to interrupt the antibiotic therapy when they start to feel healthy and to inform them about how to prevent and handle common side-effects. As associations between personality and adherence mainly have been described in relation to long-term treatments in chronic diseases, the current study add to the literature by showing that personality traits also seem to be reflected in adherence to shorter treatment periods with antibiotics for common infections. More studies in this specific area of adherence research are recommended.

## Background

Deviating from the instructions on an antibiotics prescription may lead to a flare-up of the infection, but also to the development of resistant bacteria (The World Health Organization 2003). Bacteria develop resistance as a response to the antibiotics used to treat the infection. The process of antimicrobial resistance is natural, and it is accelerated by overuse or inappropriate use of antibiotics. One consequence of this development is that common or life-threatening infections have become more difficult or sometimes impossible to treat. Therefore, the resistance could lead to prolonged illness, increased risk for complications and mortality, which in turn increases the suffering for the individual and the economic burden for the society (The World Health Organization 2012). In a global survey, non-adherence to antibiotic therapy was estimated to 22.3%, but there existed a variation across countries between 9% and 44% (Pechere, Hughes, Kardas, and Cornaglia 2007). Poor patient adherence is one of several factors causing antibiotic resistance, and as one measure, the World Health Organization (WHO) emphasizes the importance of patient education in improving adherence to antibiotic treatment regimes (2012). Strategies to improve adherence to short-term treatments, antibiotic therapy for example, such as written information about the importance of taking all the medication and personal phone calls, have been described as effective (Haynes, Ackloo, Sahota, McDonald, and Yao 2008).

In order to identify patients in need of support with adherence to long-term medication treatment in connection with various chronic diseases, we have previously explored the importance of personality to adherence behaviour (Axelsson, Brink, Lundgren, and Lötvall 2011; Axelsson, et al. 2009). Personality has been found to affect people’s thoughts, feelings and behaviour (Pervin, John, and Cervone 2008). According to the five-factor model, normal personality can be described using five broad and bipolar personality traits: Neuroticism, Extraversion, Openness to experience, Agreeableness and Conscientiousness (McCrae and Costa 2002). Moreover, these personality traits can be observed and appear to function quite similarly across cultures (McCrae et al. 2004), and in both men and women (Costa, Terracciano, and McCrae 2001). People with high scores on Neuroticism are prone to being anxious, vulnerable to stress and to having difficulties controlling their impulses and desires (Costa, and McCrae 1992). Higher scores on this personality trait have been associated with poor adherence to long-term medication treatment in individuals with various chronic diseases, for example hypertension, asthma, diabetes (Axelsson, et al. 2011), and in individuals with multiple sclerosis (Bruce, Hancock, Arnett, and Lynch 2010). The trait Agreeableness concerns interpersonal behaviour, and people with high Agreeableness scores tend to be sympathetic, altruistic and cooperative (Costa, and McCrae 1992). Higher scores on this trait have also been associated with good long-term adherence behaviour to medication treatment in individuals with various chronic disease, for example hypertension, asthma, diabetes (Axelsson, et al. 2011), and in individuals with inflammatory bowel disease (Ediger et al. 2007). Conscientiousness refers to goal-directed behaviour, and people with high Conscientiousness scores could be described as reliable, conscientious and determined (Costa, and McCrae 1992). In several studies, higher scores on this trait have also been associated with good adherence behaviour to long-term treatments for various chronic diseases (Axelsson, et al. 2011); for example in individuals undergoing renal dialysis (Christensen and Smith 1995), living with HIV (O’Cleirigh, Ironson, Weiss, and Costa 2007) and in individuals prescribed cholesterol lowering treatment (Stilley, Sereika, Muldoon, Ryan, and Dunbar-Jacob 2004).

Most studies on the relation between personality and adherence behaviour have focused on adherence in people with chronic conditions (Axelsson, et al. 2011; Axelsson, et al. 2009; Christensen and Smith 1995; Ediger, et al. 2007; O’Cleirigh, et al. 2007; Stilley, et al. 2004). Thus, we have limited knowledge about the relation between personality and adherence to short-term medication treatments, e.g., to antibiotic treatment regimes. Consequently, the aim of the current study was to explore the relation between personality and adherence behaviour in people prescribed antibiotics for common infections.

## Methods

### Participants

In 2009, 5000 inhabitants in the ages between 30–70 years in two municipalities in western Sweden were invited to participate in a cross-sectional study on the relation between personality and adherence behaviour. Contact information was provided by the Swedish Population Register and the sample was selected at random. Individuals were sent information letters inviting them to participate as well as questionnaires by mail. Two reminders were sent to non-responders. A completed and returned questionnaire was regarded as consent to participate. The response rate was 40% and a non-response analysis was conducted, which has been described in detail elsewhere. Briefly, the non-response study, based on data collected through structured telephone interviews, showed no differences relating to age and education level but some differences in personality scores were identified. Responders reported chronic disease to a greater extent than non-responders (Axelsson, et al. 2011). As part of this cross-sectional study, 445 individuals returned questionnaires which contained reports on antibiotic therapy and these form the basis of the current study.

### Data collection

The questionnaires contained questions on socio-economic status and the following items on antibiotic therapy: “During the past year, have you been prescribed antibiotics/penicillin?”, “Specify the type of infection:”, “If you took antibiotics/penicillin, how many days were you prescribed to take them?” and “How many days did you take the antibiotics/penicillin?”. The last two questions were answered by choosing among the following alternatives: <7 days, 7–10 days, and 10–14 days and >14 days. “If you did not take all the days you were prescribed, why did you stop?” The respondent could choose between the following alternatives: “Had no effect”, “Felt healthy”, “Forgot” or “Side effect”.

The Neuroticism, Extraversion, and Openness to experience Five-factor Inventory (NEO-FFI) was used to collect data on personality. The NEO-FFI consists of 60 items (12 for each personality trait) with five response alternatives, scaled 1–5 (Costa, and McCrae 1992). The Cronbach’s alpha values were: Neuroticism, 0.88; Extraversion, 0.82; Openness to experience, 0.68; Agreeableness, 0.71; Conscientiousness, 0.80, which is line with previously published values (Christensen and Smith 1995).

To collect additional data on adherence, the Medication Adherence Report Scale (MARS) was used to estimate adherence behaviour on a continuous scale. The MARS contains five items (“I forget to take them”, “I alter the dose”, “I stop taking them for a while”, “I decide to miss out a dose”, and “I take less than instructed”) scaled 1–5 with a maximum score of 25, which indicates highly adherent behaviour (Horne, and Weinman 2002). The Cronbach’s alpha value was 0.82, which is in line with previously published values (Cohen et al. 2009).

### Analysis

Socio-economic data and data from the items on antibiotic therapy were analysed using descriptive statistics, i.e. frequencies, percentages, means and standard deviations (SD). Differences between subgroups were analysed using t-tests. Any deviation from the prescribed number of days of antibiotic therapy was considered non-adherence, and a binary variable was constructed that was used in a logistic regression to identify predictors of non-adherence. Associations between personality traits and the MARS were explored using Pearson’s correlation coefficient and multiple regression (Brace, Kemp, and Snelgar 2006).

### Ethical considerations

The study was approved by the regional research ethics board at the University of Gothenburg.

## Results

Four hundred and forty-five respondents reported that they had been prescribed antibiotics for various infections (see Table 1). Deviation from the prescribed number of days of antibiotic treatment was found in 42 respondents (9.4%), 19 men and 23 women. The respondents reported that they terminated the antibiotic therapy in advance because they felt healthy (n = 19, 4.3%), they experienced side-effects (n = 12, 2.7%), they did not perceive any effect (n = 6, 1.3%) and because they forgot to take the medication (n = 5, 1.1%). Non-adherent respondents scored lower on both Agreeableness p < 0.005 (mean 43.9 SD 5.2 versus 46.4 SD 5.8) and Conscientiousness p < 0.035 (mean 44.3 SD 6.0 versus 46.4 SD 6.0) than those who did not deviate from the prescription. Non-adherent respondents also had lower scores on the MARS p < 0.001 (mean 20.1 SD 4.5 versus 23.1 SD 2.7) than adherent respondents.

**Table 1:** **Characteristics of the respondents (n = 445)**

	n (%)
**Sex**	
Men	159 (36%)
Women	286 (64%)
**Mean age (Standard deviation)**	50.8 (11.7)
**Education level**	
Compulsory school	104 (23.4%)
Upper secondary school	190 (42.7%)
University	147 (33%)
Missing	4 (0.9%)
**Own income in SEK**	
<15 000	111 (24.9%)
15 000–25 000	185 (41.6%)
25 000–40 000	105 (23.6%)
>40 000	15 (3.4%)
Missing	29 (6.5%)
**Reported infection**	
Tonsillitis	52 (11.7%)
Urinary infection	95 (21.3%)
Otitis	31 (7%)
Pneumonia	43 (9.7%)
Wound infection	44 (9.9%)
Respiratory tract infection	74 (16.6%)
**Antibiotics: days prescribed**	
<7 days	50 (11.2%)
7–10 days	266 (59.8%)
10–14	85 (19.1%)
>14	37 (8.3%)
Missing	7 (1.6%)
**Antibiotics: days taken**	
<7 days	57 (12.8%)
7–10 days	254 (57.1%)
10–14	79 (17.8%)
>14	33 (7.4%)
Missing	22 (4.9%)

A logistic analysis was performed with non-adherence to prescribed antibiotic therapy as the dependent variable and personality traits as the predictor variables. The model (chi-square 14.55, df = 5, p < 0.012) accounted for 7.0% of the variance (Nagelkerke R^2^). Table 2 shows that Agreeableness predicted non-adherence to antibiotic therapy; each unit increase in this personality trait decreased the odds of deviation from the prescribed therapy, i.e. the higher scores on Agreeableness, the lower risk for non-adherence.

**Table 2:** **Logistic regression showing odds of non-adherence to prescribed antibiotic therapy**

	Non-adherence	
Factors	Odds (C.I. 95%)	p-value
**Neuroticism**	1.012 (0.967–1.060)	0.601
**Extraversion**	1.062 (0.998–1.130)	0.060
**Openness to experience**	0.955 (0.899–1.015)	0.142
**Agreeableness**	0.924 (0.865–0.986)	0.018
**Conscientiousness**	0.951 (0.892–1.014)	0.123

C.I. = confidence interval.

Table 3 shows bivariate correlations associations between the investigated personality traits and the MARS. Neuroticism correlated negatively with the MARS, whereas Extraversion, Agreeableness and Conscientiousness correlated positively with the MARS. A multiple regression model (F = 13.042, p < 0.001), explaining 11% of the variance in MARS (Adjusted R^2^ = 0.105), identified Neuroticism as a negative predictor of adherence behaviour, i.e. higher scores on this trait predicted poorer adherence behaviour. Agreeableness and Conscientiousness were identified as positive predictors of adherence behaviour, i.e. higher scores on these traits predicted better adherence behaviour. Extraversion was not identified as a predictor of adherence behaviour in the multiple regression model (Table 3).

**Table 3:** **Pearson’s correlation coefficients (r) between MARS** ^**#**^ **and personality traits and a multiple regression model with MARS** ^**#**^ **as dependent variable and personality traits as independent variables**

	Pearson’s ***r***	Multiple regression model		
Variables	MARS^#^	B	SE B	p-values
**Neuroticism**	−.219**	−0.040	−0.115	0.049
**Extraversion**	.107*	−0.043	−0.096	0.088
**Openness to experience**	−.023	-	-	-
**Agreeableness**	.285**	0.134	0.241	0.001
**Conscientiousness**	.218**	0.068	0.135	0.017

^#^MARS = Medication Adherence Report Scale.

**p < 0.01, *p < 0.05.

## Discussion

The current study suggests that personality is of importance to adherence to short-term treatment, given that Neuroticism, Agreeableness and Conscientiousness played a role in adherence to the antibiotic therapy. Non-adherence was estimated to 9.4%, and the most common reason for prematurely stopping therapy was that the respondent felt healthy.

People with high scores on Neuroticism have a greater propensity to display worry, anxiety and vulnerability to stress. In comparison, low scorers on this personality trait are more inclined to have an even-tempered disposition (Costa, and McCrae 1992). The behavioural tendencies associated with higher scores on Neuroticism may explain the poorer adherence behaviour among the respondents in the present study. The findings are in line with previous research showing associations between higher scores Neuroticism and poorer adherence to long-term medication treatment (Bruce, Hancock, Arnett, and Lynch 2010). It is possible that adherence support for persons with higher scores on Neuroticism should address their worries.

People with high scores on Agreeableness have a propensity to be sympathetic and to cooperate (Costa, and McCrae 1992), which could explain why Agreeableness had a positive influence on adherence to antibiotic therapy in the current study and in previous research focusing adherence to long-term medication treatment for chronic disease (Ediger et al. 2007). People with low scores on this trait tend to be antagonistic, skeptical of others’ intentions and competitive instead of cooperative (Costa, and McCrae 1992). These characteristics may explain why, in the current study, low scores on this trait were associated with non-adherence. Agreeableness concerns interpersonal behaviour, and low scorers on this trait are prone to being sceptical and reluctant (Costa, and McCrae 1992). Thus, we might expect that, for this group, support intended to improve adherence to antibiotic therapy should focus on the health-care relationship and on achieving mutual trust.

There was also a significant relation between Conscientiousness and adherence to antibiotic therapy. Lower scores on this trait were found among the non-adherent respondents. Less conscientious people are less likely to actively plan and organize things and may be somewhat unreliable (Costa, and McCrae 1992), which could explain the non-adherent behaviour of respondents scoring low on Conscientiousness in the current study. Previous research has presented similar associations between this personality trait and adherence to long-term medication treatment for various chronic conditions (Christensen and Smith 1995; O’Cleirigh, Ironson, Weiss, and Costa 2007; Stilley, Sereika, Muldoon, Ryan, and Dunbar-Jacob 2004). Thus, we might expect that this group would benefit from adherence support in the form of help with developing routines for taking antibiotics.

The current results are in line with previous research showing associations between personality traits and adherence behaviour in individuals prescribed long-term medication treatments for various chronic diseases (Christensen and Smith 1995; Ediger, et al. 2007; O’Cleirigh, et al. 2007; Stilley, et al. 2004), which could be seen as a strength. However, it could be argued that assessing and taking personality into consideration is unrealistic in daily clinical practice when prescribing antibiotics for shorter treatment periods for common infections. With reference to our current understanding that inappropriate use of antibiotics could lead to an unnecessary burden for both the individual and the society (The World Health Organization 2012) every effort to increase adherence is of significance. Therefore, an increased understanding of patients’ individual differences may be one option to promote adherence. It may be unreasonable to formally assess personality traits in clinical practice but an increased awareness of patients’ needs and resources should be taken into consideration, according to the results in the current study. The results suggest that efforts to improve adherence should be matched to each patient instead of a “one-method-fits-everyone-approach”.

Personality traits do not explain all the variance in adherence behavior, which indicates that having an awareness of patients’ individual differences, is to be combined with other measures. A Cochrane review describing interventions to improve adherence concluded that measures such as personal phone calls, written information and counseling had a positive effect on adherence to short-term treatments (Haynes, Ackloo, Sahota, McDonald, and Yao 2008). The current study showed that common reasons for stopping therapy prematurely was that the respondent was now healthy and/or experienced side-effects, which indicates that for instance patient information and follow-up tailored to each patient may be recommendable to improve adherence to antibiotic therapy.

Non-adherence to antibiotic therapy in the current study was lower than the average non-adherence in other studies (Kardas, Devine, Golembesky, and Roberts 2005; Pechere, et al. 2007), although requirements for being classified as adherent were stringent. Nevertheless, it is to be noted that Pechere et al. (2007) reported that non-adherence to antibiotic therapy in some countries was almost as low as in the current study. For instance, 9.9% so called “admitted non-compliance” was found in one country. One explanation for the low non-adherence in the current study could be that patients in Sweden are made aware of the importance of taking the full course of antibiotics. Another explanation could be that this low rate could result from the adherence data being gathered by self-reports, which may suffer from bias caused by respondents’ recall or wish to project social desirability, thus leading to unduly high adherence reports (Shumaker, Ockene, and Riekert 2008). Therefore, the use of self-reports to monitor adherence could be viewed as a weakness with the current study. Another possible limitation of the current study is that the items on antibiotic use were not validated. However, they were compared with the MARS (Horne, and Weinman 2002) and the findings were similar, which could be seen as a strength.

A further limitation is the low response rate, which could have implications for the representativeness of the findings. One strength may be that the findings are based on a random population and a non-response study was conducted. To the best of our knowledge, the current study is entering a new area of adherence research by showing that personality traits not only is associated with long-term adherence to medication treatments in various chronic diseases but also seem to be of significance in relation to short-term adherence to antibiotic therapy for common infections. More studies in this research area are needed before any conclusions can be drawn.

## Conclusions

To our knowledge, this is the first study to report on the relation between personality and antibiotic therapy. The findings show that personality traits were reflected in adherence to shorter treatment periods with antibiotics for common infections. Taking individual differences into consideration could be one way of identifying individuals who need support with adherence behaviour.
